# Genome-wide interaction of genotype by erythrocyte n-3 fatty acids contributes to phenotypic variance of diabetes-related traits

**DOI:** 10.1186/1471-2164-15-781

**Published:** 2014-09-11

**Authors:** Ju-Sheng Zheng, Chao-Qiang Lai, Laurence D Parnell, Yu-Chi Lee, Jian Shen, Caren E Smith, Patricia Casas-Agustench, Kris Richardson, Duo Li, Sabrina E Noel, Katherine L Tucker, Donna K Arnett, Ingrid B Borecki, José M Ordovás

**Affiliations:** Jean Mayer USDA Human Nutrition Research Center on Aging at Tufts University, Boston, MA USA; Department of Food Science and Nutrition, Zhejiang University, Hangzhou, China; Bone & Mineral Unit, Division of Endocrinology, Oregon Health & Science University, Portland, OR USA; Department of Clinical Laboratory & Nutritional Sciences, University of Massachusetts Lowell, Lowell, MA USA; Department of Epidemiology, University of Alabama at Birmingham, Birmingham, AL USA; Department of Genetics, Washington University School of Medicine, St. Louis, MO USA; Instituto Madrileño de Estudios Avanzados Alimentación, Ciudad Universitaria de Cantoblanco, Madrid, Spain

## Abstract

**Background:**

Little is known about the interplay between n-3 fatty acids and genetic variants for diabetes-related traits at the genome-wide level. The present study aimed to examine variance contributions of genotype by environment (GxE) interactions for different erythrocyte n-3 fatty acids and genetic variants for diabetes-related traits at the genome-wide level in a non-Hispanic white population living in the U.S.A. (n = 820). A tool for Genome-wide Complex Trait Analysis (GCTA) was used to estimate the genome-wide GxE variance contribution of four diabetes-related traits: HOMA-Insulin Resistance (HOMA-IR), fasting plasma insulin, glucose and adiponectin. A GxE genome-wide association study (GWAS) was conducted to further elucidate the GCTA results. Replication was conducted in the participants of the Boston Puerto Rican Health Study (BPRHS) without diabetes (n = 716).

**Results:**

In GOLDN, docosapentaenoic acid (DPA) contributed the most significant GxE variance to the total phenotypic variance of both HOMA-IR (26.5%, *P*-nominal = 0.034) and fasting insulin (24.3%, *P*-nominal = 0.042). The ratio of arachidonic acid to eicosapentaenoic acid + docosahexaenoic acid contributed the most significant GxE variance to the total variance of fasting glucose (27.0%, *P*-nominal = 0.023). GxE variance of the arachidonic acid/eicosapentaenoic acid ratio showed a marginally significant contribution to the adiponectin variance (16.0%, *P*-nominal = 0.058). None of the GCTA results were significant after Bonferroni correction (*P* < 0.001). For each trait, the GxE GWAS identified a far larger number of significant single-nucleotide polymorphisms (*P*-interaction ≤ 10E-5) for the significant E factor (significant GxE variance contributor) than a control E factor (non-significant GxE variance contributor). In the BPRHS, DPA contributed a marginally significant GxE variance to the phenotypic variance of HOMA-IR (12.9%, *P*-nominal = 0.068) and fasting insulin (18.0%, *P*-nominal = 0.033).

**Conclusion:**

Erythrocyte n-3 fatty acids contributed a significant GxE variance to diabetes-related traits at the genome-wide level.

**Electronic supplementary material:**

The online version of this article (doi:10.1186/1471-2164-15-781) contains supplementary material, which is available to authorized users.

## Background

Diabetes has become one of the most common chronic diseases, with the world prevalence among adults of 6.4% in 2010. It has been estimated that this figure will increase to 7.7% by 2030, with the absolute number increasing from 285 to 439 million [1]. Type 2 diabetes (T2D) accounts for more than 90% of diabetes cases and is characterized by insulin resistance and impaired β-cell function. Accumulating evidence suggests that T2D is a result of complex interplay between genetic and environmental factors [2], supporting the notion that a healthier lifestyle may attenuate the adverse effects of T2D risk alleles, while an unhealthy lifestyle may augment it. Dietary n-3 polyunsaturated fatty acids (PUFA) have been demonstrated in rodents to increase insulin sensitivity through a number of different mechanisms, including anti-inflammatory effects, regulation of circulating hormones and transcription factors, with effects on membrane fluidity and improvements in lipid profiles [3]. In contrast, evidence from observational studies [4] and clinical trials [5] in humans shows inconsistent association between n-3 PUFA and insulin sensitivity and T2D. These discrepancies may be attributed partly to the influence of genotype by environment (GxE) interactions with regard to n-3 PUFA [6, 7].

Different n-3 PUFAs may affect insulin sensitivity and insulin secretion through different mechanisms [3, 8]. In a human randomized controlled trial [9], eicosapentaenoic acid (EPA, C20:5n3) and docosahexaenoic acid (DHA, C22:6n3), the two major n-3 PUFA, showed differential effects on glucose metabolism. In addition, the effect of alpha-linolenic acid (ALA, C18:3n3) on insulin resistance and T2D differs from that of DHA and EPA [8]. Thus, it can be postulated that the patterns for the interplay between n-3 PUFA intake and the genome varies by type and intake level. However, studies characterizing the GxE interaction of different types of n-3 PUFA with genotypes on T2D-related traits are sparse. Our previous study demonstrated the importance of genome-wide GxE interactions in explaining phenotypic variance of diabetic traits [10]. To date, no published data are available as to what percentage of total variance of a given T2D-related trait can be explained by GxE interactions of individual n-3 PUFA with the whole genome.

There were three primary objectives of the present study. One, as erythrocyte membrane n-3 PUFAs are widely accepted biomarkers for the dietary n-3 PUFA intake, we sought to demonstrate the role of the GxE interaction between different types of erythrocyte n-3 PUFAs and genotypes for the variation of T2D-related traits at the genome-wide level. Two, we explored the extent of these GxE variance contributions in a non-Hispanic white population living in the U.S.A (Table 1). Three, for a given T2D-related trait, our study sought to identify a corresponding n-3 PUFA that contributed the most significant GxE variance for each trait. Replication was conducted in a Puerto Rican population living in the U.S.A.

**Table 1:** **Population characteristics of the GOLDN study and participants without diabetes in the BPRHS** ^**1**^

	GOLDN (n = 820)			BPRHS (n = 716)		
	Mean	SD	Range (Q1-Q3)	Mean	SD	Range (Q1-Q3)
Age, y	48.9	16	39-62	56.0	7.53	50-61
Female, n (%)	414 (50.5)			501 (70.0)		
BMI, kg/m^2^	28.5	5.5	24.8-31.4	30.6	6.27	26.3-34.0
Energy intake, kJ	8942	5259	5926-10684	10051	5523	6343-11998
Fasting glucose, mmol/L	5.66	1.09	5.11-5.88	5.41	0.61	5.00-5.83
Fasting insulin, pmol/L	97.7	57.4	62.5-111.1	99.7	65.4	57.6-122.2
HOMA-IR	3.61	2.48	2.15-4.2	3.54	2.60	1.92-4.38
Adiponectin, ng/mL	8109	4500	4664-10210	NA	NA	NA
Erythrocyte n-3 PUFA,%	5.78	1.1	5.00-6.33	6.47	1.37	5.60-7.08
Erythrocyte n-6 PUFA,%	28.2	1.5	27.4-29.2	31.0	2.19	30.1-32.3
Erythrocyte DHA,%	3.01	0.86	2.37-3.46	3.91	1.05	3.23-4.51
Erythrocyte EPA,%	0.52	0.23	0.39-0.59	0.42	0.23	0.29-0.49
Erythrocyte DPA,%	2.11	0.29	1.93-2.27	2.00	0.35	1.79-2.19
Erythrocyte ALA,%	0.14	0.03	0.11-0.16	0.13	0.06	0.09-0.16
Erythrocyte AA,%	13.6	1.1	12.9-14.4	16.5	1.84	15.5-17.6
(n-6)/ (n-3) PUFA	5.06	0.98	4.39-5.74	5.02	1.17	4.30-5.58
AA/ (EPA + DHA)	4.14	1.11	3.36-4.85	4.07	1.13	3.32-4.71
AA/ DHA	4.89	1.41	3.86-5.78	4.51	1.28	3.65-5.24
AA/ EPA	29.6	10.1	22.8-35.9	46.9	19.5	33.6-56.7

^1^BMI, body mass index; SD, standard deviation; Q, quartile; HOMA-IR, homeostasis model assessment of insulin resistance; PUFA, polyunsaturated fatty acid; DHA, docosahexaenoic acid; EPA, eicosapentaenoic acid; DPA, docosapentaenoic acid; ALA, alpha-linolenic acid; AA, arachidonic acid.

## Results

### Genome-wide GxE variance contribution to the phenotypic variance of T2D traits

**Figure 1:**
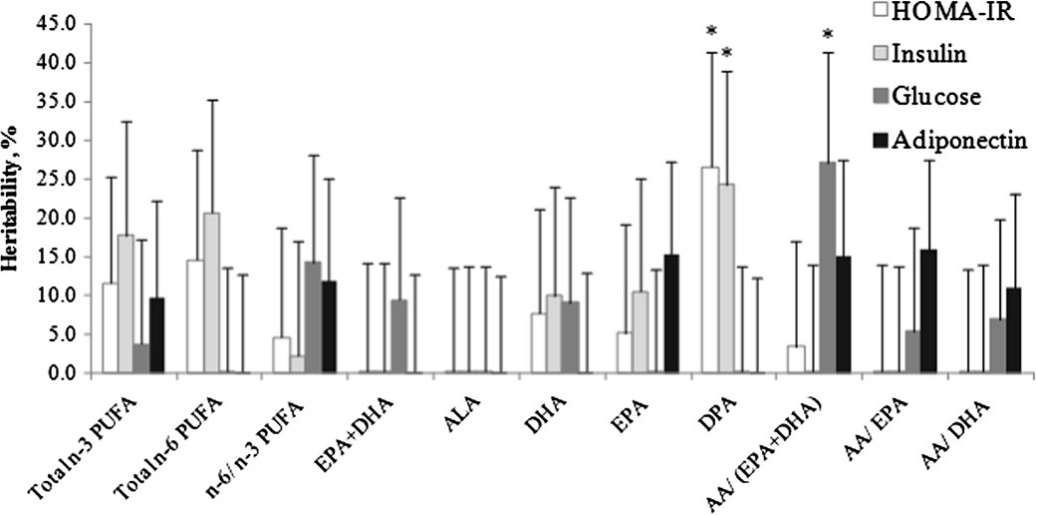
**GxE variance estimation of n-3 polyunsaturated fatty acids for four diabetes-related traits.** The GxE variance is shown as the percentage of the total phenotypic variance of each trait (heritability). *P < 0.05 indicates significant contribution to total variance. Data are expressed as mean ± SE.

**Figure 2:**
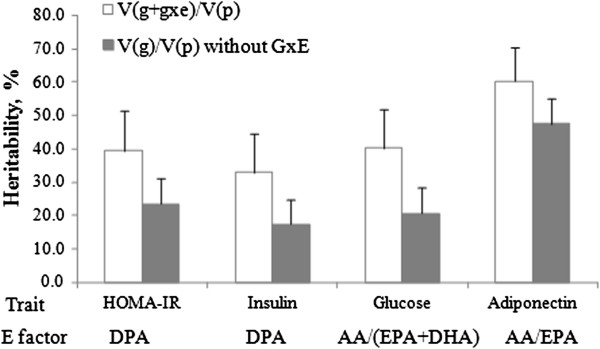
**Estimated heritability (%) of diabetes-related traits.** Solid bars depict the heritability based on additive genetic variance. Unfilled bars represent heritability, as a percentage, arising from the sum of additive genetic variance and genetic variance by GxE interaction. GxE heritability was calculated as the GxE variance divided by the total phenotypic variance. DPA, docosapentaenoic acid; AA, arachidonic acid; EPA, eicosapentaenoic acid; DHA, docosahexaenoic acid. Data are expressed as mean ± SE.

For each trait, GCTA was conducted in GOLDN to assess the genome-wide variance contribution of genotype by erythrocyte n-3 PUFAs to total phenotypic variance. When the GxE was not included in the models, additive genetic variance contribution for HOMA-IR, fasting insulin, glucose and adiponectin was 23.4% (*P*-nominal = 4.5 × 10^-4^), 17.3% (*P*-nominal =5.3 × 10^-3^), 20.6% (*P*-nominal = 1.9 × 10^-3^), and 47.5% (*P*-nominal = 4.5 × 10^-13^), respectively. When the GxE term was included in the models for each HOMA-IR and fasting insulin, only DPA contributed a significant GxE variance to the total phenotypic variance (26.5% for HOMA-IR, *P*-nominal = 0.034; 24.3% for fasting insulin, *P*-nominal = 0.042) (Table 2, Figures 1 and 2). The additive genetic variance ranged from 12.8% to 21.3% for HOMA-IR depending on the PUFA E factors (Additional file 1: Table S1), and from 8.6% to 16.0% for fasting insulin (Additional file 1: Table S2). For fasting glucose, 27.0% of the total phenotypic variance (*P*-nominal = 0.023) was explained by the GxE of the ratio of AA/(EPA + DHA), whereas 13.1% of the variance was accounted for by additive genetic variance (Table 2, Additional file 1: Table S3). The contributions of other E factors to the variance of these traits did not reach statistical significance. None of the GCTA results were significant after Bonferroni correction (*P* < 0.001).

**Table 2:** **GxE variance contribution of erythrocyte n-3 polyunsaturated fatty acids to four diabetes-related traits in GOLDN** ^**1**^

Trait	E factor	***P***-nominal (gxe) ^2^	Vg	SE	V(gxe)	SE	h ^2^ (g), % (95% CI)	SE	h ^2^ (gxe), % (95% CI)	SE	h ^2^ (g + gxe), % (95% CI)
HOMA-IR	DPA	0.034	0.0007	0.0005	0.0015	0.0008	12.8 (0, 30.6)	9.1	26.5 (0, 55.7)	14.9	39.4 (15.9, 62.9)
Fasting insulin	DPA	0.042	0.0002	0.0002	0.0006	0.0004	8.6 (0, 25.8)	8.8	24.3 (0, 53.1)	14.7	32.9 (9.87, 55.9)
Fasting glucose	AA/ (EPA + DHA)	0.023	0.4518	0.3076	0.9295	0.4979	13.1 (0, 30.3)	8.8	27.0 (0, 55.0)	14.3	40.2 (17.6, 62.8)
Adiponectin	AA/ EPA	0.058	0.0200	0.0044	0.0072	0.0052	44.4 (27.3, 61.5)	8.7	16.0 (0, 38.3)	11.4	60.4 (40.7, 80.1)

^1^HOMA-IR, HOMA-Insulin Resistance; DHA, docosahexaenoic acid; EPA, eicosapentaenoic acid; DPA, docosapentaenoic acid; ALA, alpha -linolenic acid; AA, arachidonic acid;Vg, additive genetic variance; V(gxe), variance contributed by GxE interaction; SE, standard error; h^2^ (g), additive genetic heritability; h^2^ (gxe), heritability explained by GxE interaction; h^2^ (g + gxe), total heritability. GxE heritability was calculated as the GxE variance divided by the total phenotypic variance. Only the nominal significant E factors were listed in this table, results of other E factors were listed in the Additional files 1 and 2.

^2^ *P*-value (gxe) of GxE interaction was adjusted for age, sex, body mass index, study center, energy intake, kinship, and population structure.

T2D is closely associated with inflammation and is considered an inflammatory disease [11]. Adiponectin is well known for its anti-inflammatory effects in several tissues [12], and recent evidence suggests that it is associated with T2D risk [13]. Therefore, we treated fasting plasma adiponectin as a T2D-related trait and tested the GxE variance contribution to this trait. For adiponectin, the AA/ EPA ratio in GOLDN contributed a marginally significant GxE variance (16.0%, *P*-nominal = 0.058) (Table 2, Figures 1 and 2). Although only marginally significant, the variance of adiponectin explained by the additive genetic variance was stable in different GxE models, ranging from 44.4% to 48.9% (Additional file 1: Table S4).

To test whether the significant GxE variance contribution of one E factor was affected by the other E factors, we used data from GOLDN to pair the nominally significant E factor with another E factor simultaneously in the GCTA model (Table 3). For example, for both HOMA-IR and fasting insulin, we paired DPA with total n-3 PUFA, n-6 PUFA or DHA in the model. Inclusion of either E factor did not alter the nominally significant GxE variance contribution of DPA. We paired the AA/(EPA + DHA) ratio with total n-3/n-6 ratio or EPA + DHA for fasting glucose, and consistently, the nominally significant contribution of GxE variance of AA/(EPA + DHA) ratio did not change. However, for adiponectin, the *P*-values for the GxE contribution of both EPA and the AA/EPA ratio were attenuated when they were included in the same model, due to the high correlation between erythrocyte measures of AA/EPA and EPA (r = -0.97, *P* < 0.001). As proxies for long-term dietary intakes, both factors represented the same GxE variance for adiponectin.

**Table 3:** **Estimation of GxE variance for paired environmental factors on four diabetes-related traits in GOLDN** ^**1**^

Trait	E factor	h ^2^ (g)	SE	h ^2^ (gxe)	SE	***P***-nominal ^2^
HOMA-IR	DPA	0.100	0.099	0.264	0.149	0.034
	n-3 PUFA			0.096	0.135	0.225
	DPA	0.067	0.103	0.291	0.149	0.023
	n-6 PUFA			0.194	0.138	0.069
	DPA	0.124	0.099	0.250	0.149	0.044
	DHA			0.052	0.134	0.342
Fasting insulin	DPA	0.050	0.097	0.242	0.146	0.041
	n-3 PUFA			0.141	0.144	0.161
	DPA	0.025	0.099	0.261	0.146	0.032
	n-6 PUFA			0.243	0.144	0.039
	DPA	0.076	0.097	0.228	0.146	0.053
	DHA			0.066	0.139	0.312
Fasting glucose	AA/ (DHA + EPA)	0.127	0.091	0.264	0.160	0.043
	n-6/ n-3 PUFA			0.018	0.150	0.450
	AA/ (DHA + EPA)	0.112	0.090	0.279	0.163	0.032
	DHA + EPA			0.000	0.150	0.500
Adiponectin	AA/ EPA	0.447	0.088	0.096	0.151	0.265
	EPA			0.078	0.157	0.318
	AA/ EPA	0.419	0.096	0.159	0.115	0.061
	AA/ DHA			0.094	0.122	0.210

^1^For each trait, one significant E factor (significant GxE variance contributor) was simultaneously paired with another E factor into the model, and corresponding GxE variance for either E factor was estimated in the model. GxE heritability was calculated as the GxE variance divided by the total phenotypic variance. PUFA, polyunsaturated fatty acid; DHA, docosahexaenoic acid; EPA, eicosapentaenoic acid; DPA, docosapentaenoic acid; ALA, alpha-linolenic acid; AA, arachidonic acid; SE, standard error; h^2^(g), heritability of additive genetic variance; h^2^ (gxe), heritability of GxE interaction.

^2^ *P*-values were adjusted for age, sex, body mass index, study center, energy intake, kinship, and population structure.

### GxE interaction of n-3 PUFAs at the genome-wide level

**Figure 3:**
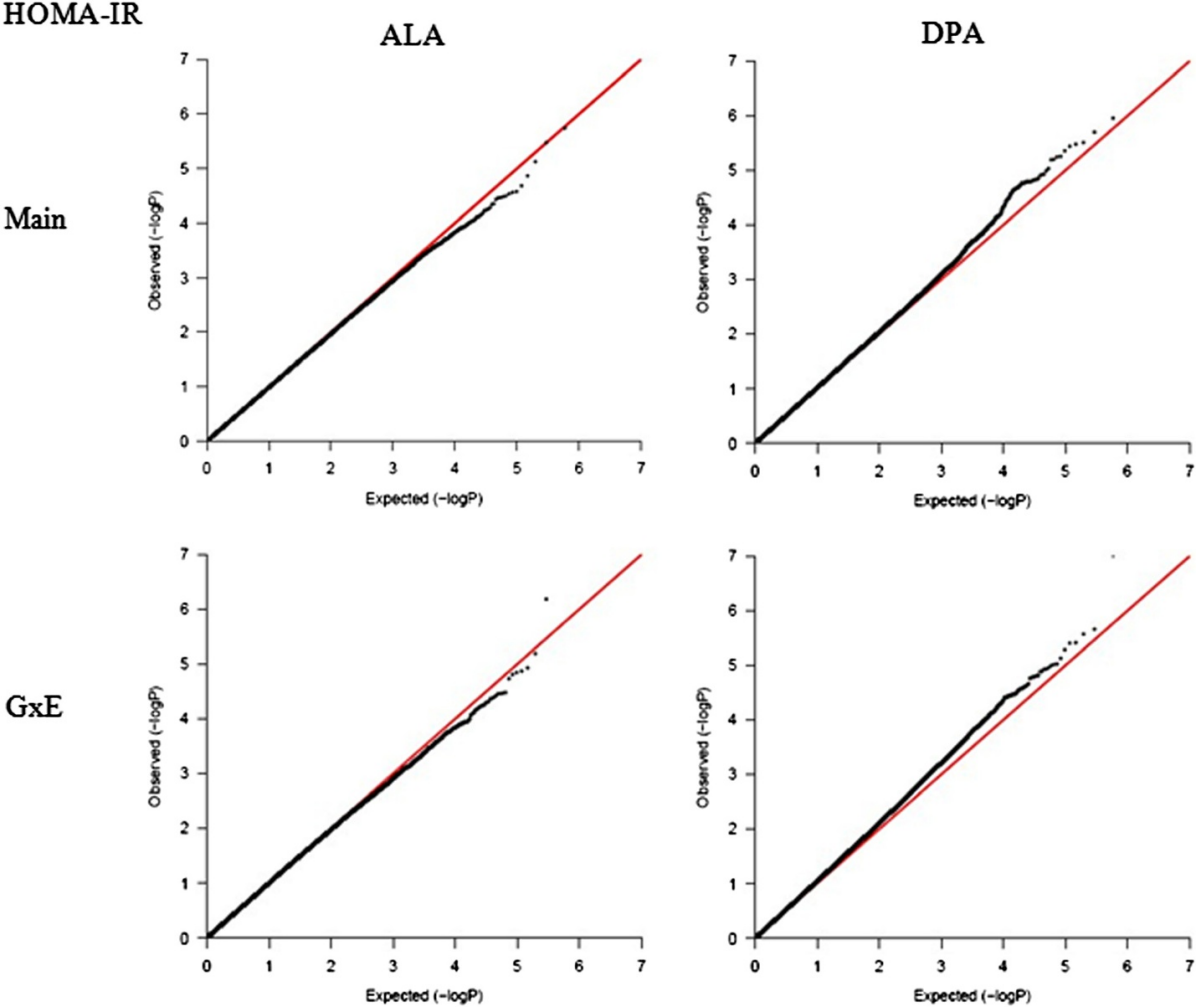
**QQ-plot for HOMA-IR.** Two plots on the left are for the main effect and GxE interaction of ALA (control E factor), while two plots on the right are for the DPA, which contributed a nominal significance to the GxE variance.

**Figure 4:**
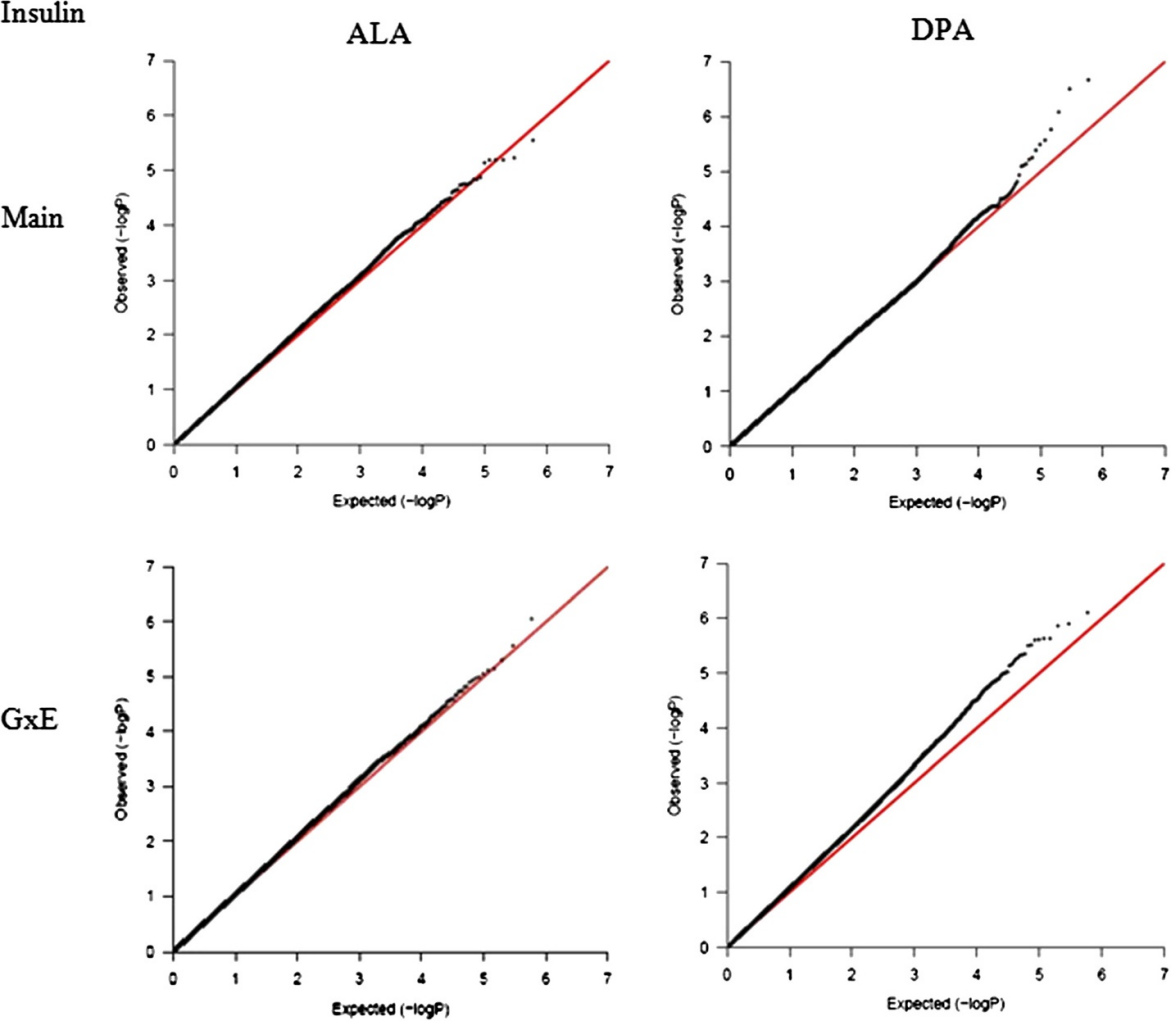
**QQ-plot for fasting insulin.** Two plots on the left are for the main effect and GxE interaction of ALA (control E factor), while two plots on the right are for the DPA, which contributed a nominal significance to the GxE variance.

**Figure 5:**
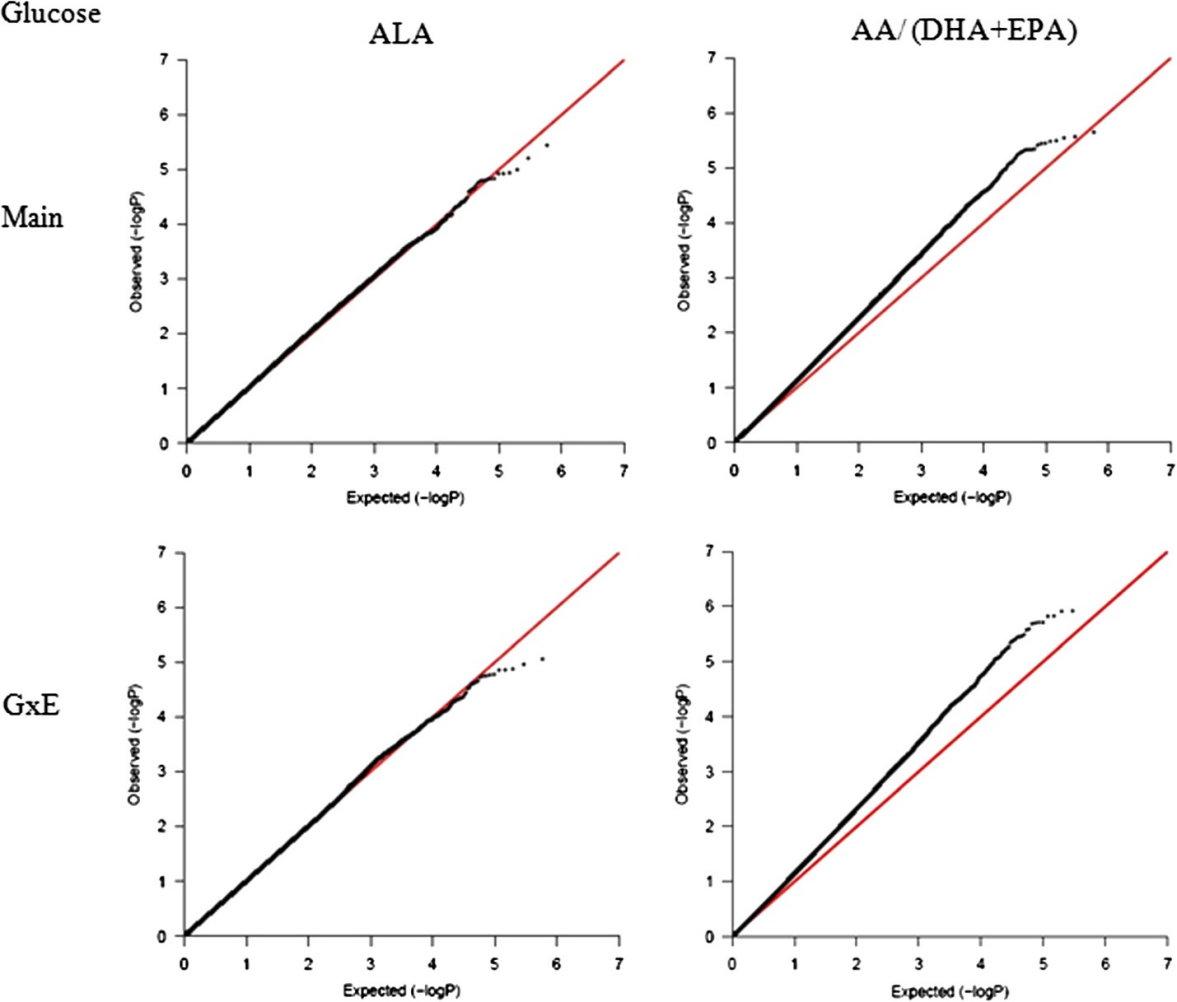
**QQ-plot for fasting glucose.** Two plots on the left are for the main effect and GxE interaction of ALA (control E factor), while two plots on the right are for the AA/(DHA + EPA), which contributed a nominal significance to the GxE variance.

**Figure 6:**
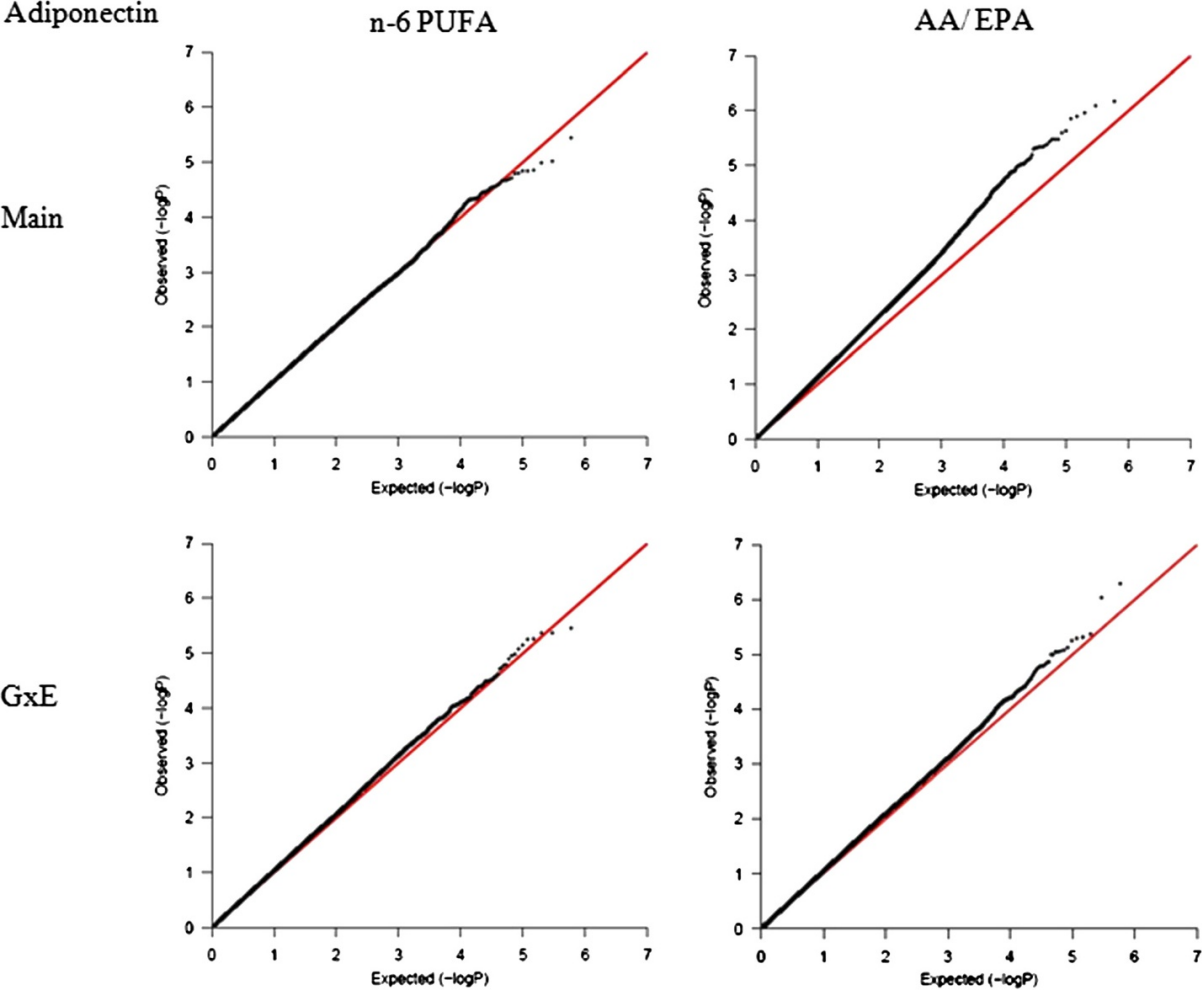
**QQ-plot for fasting adiponectin.** Two plots on the left are for the main effect and GxE interaction of n-6 PUFA (control E factor), while two plots on the right are for the AA/EPA, which contributed a nominal significance to the GxE variance.

To illustrate further the significance of the contribution of G×E interaction of PUFA on diabetes-related phenotypes, a G×E GWAS was conducted in GOLDN for the GCTA-identified significant E factor using the GWAF (Table 4, Additional file 2: Table S5). For each trait, we identified one E factor, which contributed a nominally significant GxE variance using GCTA, and another E factor, which contributed a non-significant GxE variance, serving as control. For HOMA-IR, the GxE GWAS of DPA identified nine significant SNPs (*P*-interaction ≤ 1.0 × 10^-5^), but just three for ALA, the control E factor. Of greater interest, the GxE GWAS of DPA for fasting insulin identified 21 significant SNPs but only six for the control E factor (ALA). Similarly, the GCTA-identified significant E factors for fasting glucose and for adiponectin each produced more significant SNPs in the GxE GWAS, compared with their corresponding control E factors. The number of GxE GWAS-identified SNPs (*P*-interaction ≤ 1.0 × 10^-5^) for fasting glucose and its significant E factor AA/(EPA + DHA) was 36, with just a single SNP identified for the corresponding control E factor. For adiponectin, there were 13 GxE GWAS-identified SNPs (*P*-interaction ≤ 1.0 × 10^-5^) with the AA/EPA as the significant E factor, and 7 for the corresponding control E factor. For either trait, the QQ-plot drawn based on the *P*-values of GxE interaction were slightly above the diagonal line for the E factor, which contributed a significant G×E variance, while for the control E factor, the QQ-plot aligned well with the diagonal line (Figures 3, 4, 5 and 6). These QQ-plots confirm the GCTA results.

**Table 4:** **The number of SNPs with** ***P*** **-value <1 × 10** ^**-5**^ **based on GxE GWAS in GOLDN** ^**1**^

	Without GxE			With GxE		
Trait ^2^	Main effect	E factor	GxE ^3^	Main effect	GxE interaction	Total for main and GxE
HOMA-IR	5	DPA	S	11	9	20
		ALA	NS	3	3	6
Fasting insulin	7	DPA	S	12	21	32
		ALA	NS	6	6	12
Fasting glucose	9	AA/ (EPA + DHA)	S	25	36	57
		ALA	NS	2	1	3
Adiponectin	18	AA/ EPA	S	33	13	44
		n-6 PUFA	NS	2	7	9

^1^PUFA, polyunsaturated fatty acid; DHA, docosahexaenoic acid; EPA, eicosapentaenoic acid; DPA, docosapentaenoic acid; ALA, alpha -linolenic acid; AA, arachidonic acid; GWAS, genome-wide association study.

^2^For all these traits, GWAS and GxE GWAS were adjusted for age, sex, body mass index, study center, energy intake, kinship, and population structure.

^3^The E factor has a significant (S) or non-significant (NS) GxE variance contribution to the total phenotypic variance of either diabetes-related trait based on GxE GWAS.

### Replication of GxE variance contribution to the phenotypic variance of T2D traits in participants of the BPRHS without diabetes

In the BPRHS, when the GxE term was not included in the models, the additive genetic variance contribution for HOMA-IR, fasting insulin and glucose was 8.83% (*P*-nominal = 0.02), 6.24% (*P*-nominal = 0.06) and 13.8% (*P*-nominal = 0.006), respectively. Including the GxE term in the models showed that DPA contributed a marginally significant GxE variance to the phenotypic variance of HOMA-IR (12.9%, *P*-nominal = 0.068) and fasting insulin (18.0%, *P*-nominal = 0.033) (Table 5). However, no significant GxE variance contribution of the ratio of AA/(EPA + DHA) for fasting glucose variation was observed. For the control E factor (ALA), no significant GxE variance contribution was observed for any of the three traits (*P*-nominal ≥ 0.50).

**Table 5:** **Replication of GxE variance contribution of erythrocyte n-3 polyunsaturated fatty acids to diabetes-related traits in the BPRHS** ^**1**^

Trait	E factor	***P***-nominal (gxe) ^2^	Vg	SE	V(gxe)	SE	h ^2^ (g), % (95%CI)	SE	h ^2^ (gxe), % (95%CI)	SE	h ^2^ (g + gxe), % (95%CI)
HOMA-IR	DPA	0.068	0.0150	0.0300	0.0446	0.0451	4.34 (0, 21.3)	8.66	12.9 (0, 38.4)	13.0	17.1 (0, 38.3)
	ALA	0.500	0.0350	0.0350	0	0.019	10.0 (0, 29.2)	9.8	0 (0, 10.5)	5.35	10.0 (0, 24.8)
Fasting insulin	DPA	0.033	0.0034	0.0232	0.0531	0.0463	1.16 (0, 16.6)	7.89	18.0 (0, 48.6)	15.6	19.2 (0, 42.2)
	ALA	0.500	0.0222	0.0251	0	0.0165	7.45 (0, 23.9)	8.40	0 (0, 10.8)	5.52	7.45 (0, 21.1)
Fasting glucose	AA/ (EPA + DHA)	0.252	0.0016	0.0015	0.0004	0.0007	13.3 (0, 38.0)	12.6	3.09 (0, 15.1)	6.14	16.4 (0, 34.8)
	ALA	0.500	0.0019	0.0017	0	0.0005	16.2 (0, 43.4)	13.9	0 (0, 9.07)	4.63	16.2 (0, 34.4)

^1^DPA, docosapentaenoic acid; ALA, alpha -linolenic acid; Vg, additive genetic variance; V(gxe), variance contributed by GxE interaction; SE, standard error; h^2^ (g), additive genetic heritability; h^2^ (gxe), heritability explained by GxE interaction; h^2^ (g + gxe), total heritability. GxE heritability was calculated as the GxE variance divided by the total phenotypic variance. ALA was served as a control.

^2^ *P*-value (gxe) of GxE interaction was adjusted for age, sex, body mass index, energy intake and population structure.

## Discussion

While it is increasingly recognized that the interplay between genetic and environmental factors contributes to T2D risk [2, 6, 7], inconsistencies among studies often obscure the importance of GxE interactions. The effects of n-3 PUFAs on chronic disease and related intermediate phenotypes, for example, have been studied throughout the last five decades, but discrepancies still exist with regard to outcomes involving human studies [7]. One of these divergences is the association between n-3 PUFA and T2D phenotypes [3–5]. Apart from the methodological differences among studies, the influence of genetic variation is likely an important contributor. Previous studies have identified numerous candidate genes that may have an influence on the effects of n-3 PUFA on T2D or related traits [7]. Recent advances in GWAS provide the opportunity to explore the influence of n-3 PUFA GxE on T2D and related traits at the genome-wide level. In the present study, we used a new method to illustrate to what extent the GxE of n-3 PUFA contributed to the variations of T2D-related traits and to characterize the most important GxE variance contributor among these fatty acids at the genome level. This approach, for the first time, gives a more detailed depiction of the interplay between different types of n-3 PUFA and a set of ~590,000 genetic variants, and their influence on the T2D-related phenotypes. In addition, we were able to distinguish the different GxE patterns for different n-3 PUFA in red blood cells, which provides a solid rationale for future research on the impact of different n-3 PUFAs with regard to disease risk and health maintenance in humans.

Consistent results from rodent models support the anti-diabetic effects of marine n-3 PUFA [3, 8], while in humans most prospective cohort studies conducted in Western countries have found null or even positive associations between n-3 PUFA and risk of T2D [4, 14]. In a meta-analysis of these cohort studies, we suggested that genetic and GxE interactions may contribute to these inconsistent associations in Western populations [4]. Previous studies, most of which employed a candidate gene approach, have identified a number of genetic variants showing GxE with n-3 PUFA on T2D-related traits [6, 7]. However, given the powerful regulatory effects of n-3 PUFAs, candidate gene approaches can provide only a narrow view of possible GxE interactions for n-3 PUFAs. Overcoming this restriction, our genome-wide studies with GCTA have supplied much information on the interaction of erythrocyte membrane n-3 PUFAs on T2D-related traits. We further illustrated the GCTA results by conducting a GxE GWAS.

Different n-3 PUFAs, such as ALA, EPA, DPA and DHA, likely exert their health-related effects through different mechanisms. Among these fatty acids, the literature regarding the biological effects of DPA is relatively limited [15]. DPA is an intermediate product of fatty acid metabolism between EPA and DHA, and available evidence suggests that DPA has both unique as well as overlapping actions compared to DHA and EPA. *In vivo* and *in vitro* studies [15] have indicated that the biological effects of DPA mainly stemmed from its effects on eicosanoid production, endothelial cell migration and gene expression related to lipogenic and inflammatory genes. However, to our knowledge, studies characterizing the effects of DPA on T2D-related traits are sparse and no published reports are available with regard to the GxE of DPA with genetic variants on T2D traits. Erythrocyte DPA contributed a nominally significant GxE variance to the total variance of HOMA-IR and fasting insulin at the genome-wide level. These results did not change when we paired DPA with other erythrocyte membrane E factors, such as total n-3 PUFA, total n-6 PUFA or DHA, in the GCTA model. This indicated that the DPA GxE variance contribution was independent of these E factors.

It is known that phenotypic flexibility decreases with age and onset of pre-clinical conditions. When the environment (eg, diet) is perturbed, there is reduced ability to identify susceptibility variants by GWAS, implying a widespread influence for GxE interactions on phenotypic variance [16]. Thus, although the two populations examined here are subject to different environmental influences, some of the GxE interactions we have identified are biologically plausible. For example, one SNP (rs10074889) near *PPARGC1B* interacted with DPA for both HOMA-IR and fasting insulin. *PPARGC1B* encodes peroxisome proliferator-activated receptor (PPAR) gamma co-activator 1-beta, and is a transcriptional co-factor contributing to the regulation of fat oxidation, energy expenditure and glucose metabolism [17]. Furthermore, *PPARGC1B* expression is down-regulated in skeletal muscle of T2D patients [18]. Therefore, it can be postulated that DPA regulates *PPARGC1B* expression and subsequently affects glucose metabolism in an allele-specific manner. Another example shows that six of the 21 GxE GWAS-identified SNPs for fasting insulin locate near the *CRPP1* and *CRP* genes, indicating that DPA may regulate insulin concentrations through an effect on C-reactive protein and inflammatory pathways [11, 19]. However, future research is warranted to characterize the functions of these identified SNPs and to explore the precise mechanism for the effect of DPA on insulin resistance and glucose metabolism.

Other interesting findings include the AA/(EPA + DHA) ratio contribution of a nominally significant GxE variance for fasting glucose, and the AA/EPA ratio contribution of a marginally significant GxE variance for adiponectin in GOLDN, which was not replicated in a second population. As indicated [20], important roles for the n-6/n-3 PUFA ratio in coronary artery disease, hypertension and T2D, diseases affected by chronic inflammation. AA is the principle precursor for eicosanoid production, including 2-series prostaglandins and 4-series leukotrienes, and these products are highly active agents of inflammation [21]. In contrast, both EPA and DHA have anti-inflammatory effects and inhibit the production of those pro-inflammatory biomarkers [3, 21]. Adiponectin is an anti-inflammatory biomarker, and thus sensitive to changes in the n-6/n-3 PUFA ratio. Our results indicate that the n-6/n-3 PUFA ratio affects the genetic susceptibility of fasting glucose and adiponectin. Combining genetic additive variance and GxE variance of the n-6/n-3 ratio explained more phenotypic variation of these two traits than genetic effects alone. Nevertheless, the results for adiponectin should be interpreted with caution because none of the GxE variance contribution of adiponectin reached statistical significance.

The GCTA method of estimating the GxE variance contribution enables us to view the pattern of the interplay between n-3 PUFA and the genome, and to re-examine the different roles of individual n-3 PUFAs or the related n-6/n-3 ratio in human health and disease. However, some limitations are present in this study. First, overestimation of the genetic and GxE variance may exist given the family-based nature of the GOLDN population, including their shared environments within family and causal variants captured by pedigree, but not by SNPs [22, 23]. This is also the reason why the estimates of heritability are generally higher in GOLDN than in BPRHS, a population comprised of unrelated individuals. Second, the sample size of the present study is moderate. Studies with larger sample size and populations of different ethnicities, but equally deeply phenotyped, are needed to confirm our results. Third, none of our primary GCTA results was significant after Bonferroni correction for multiple testing. However, the Bonferroni correction is very conservative and may not be the most appropriate correction method to apply in this circumstance. Nevertheless, our results from GCTA were further confirmed by a GxE GWAS and replicated in part (by GCTA) in a second population.

## Conclusions

We used a GCTA method to explore the GxE variance contribution of erythrocyte membrane n-3 PUFA to the variation of T2D-related traits at the genome-wide level. We demonstrated that, at the genome-wide level, different types of n-3 PUFA can contribute different GxE variances to the same phenotype, and the same n-3 PUFA contributes different amounts of GxE variances to different phenotypes. These results indicate the importance of the GxE of n-3 PUFAs for major diabetes traits, and also suggest the extent to which these GxE interactions contribute to the variation of each trait. Our results have important implications for public health research in that long-chain n-3 PUFAs have been accepted widely as dietary supplements around the world, while the effects of n-3 PUFAs on major chronic diseases, such as T2D and cardiovascular disease, remain inconclusive. The genome-wide influence of n-3 PUFA GxE explains some of these inconsistencies, and future dietary recommendations should consider the effects of genetic variation, especially as personalized nutrition gains acceptance. Furthermore, a mechanism for the unique function of each individual n-3 PUFA warrants more comprehensive research.

## Methods

### Subjects

The Genetics of Lipid Lowering Drugs and Diet Network (GOLDN) Study was designed to examine genetic factors that regulate dietary and fenofibrate responses, and the study details have been described [24, 25]. Caucasian families with at least 2 siblings of 3-generations were recruited from two centers of the National Heart, Lung, and Blood Institute Family Heart Study in Minneapolis, MN, and Salt Lake City, UT. Exclusion criteria include age < 18 years, fasting triglycerides > 16.5 mmol/L, recent history of myocardial infarction, liver, kidney, pancreas, or gall bladder disease, history of malabsorption of nutrients, current use of insulin, abnormal renal or hepatic function, and pregnancy or nursing in women. The current study examined the data of T2D-related traits collected at the baseline of the GOLDN population. The original sample size for the GOLDN study was approximately 1200, while completed biochemical data were available from 1118 individuals. Genome-wide genotyping data was obtained from 820 subjects. Baseline characteristics of the 820 GOLDN participants are shown in Table 1. All participants gave informed consent. The study protocol was approved by the Institutional Review Boards at the University of Alabama, University of Minnesota, University of Utah, and Tufts University.

Replication was conducted in the Boston Puerto Rican Health Study (BPRHS), a longitudinal cohort study of stress, nutrition, health, and aging in Puerto Ricans living in the Boston metropolitan area [26]. Erythrocyte fatty acid and genome-wide genotyping data were obtained from 1198 participants. Of these, 716 participants without diabetes were included in the final analysis (482 with diabetes were excluded) as the variance contribution analysis may be influenced by diabetes status or anti-diabetes medication (Table 1). All participants gave informed content. The study protocol was approved by the Institutional Review Boards at Tufts University and Northeastern University.

### Biochemical measurement and erythrocyte fatty acid determination

For GOLDN, venous blood was drawn after an overnight fast. Measurements of fasting plasma glucose, insulin, and adiponectin have been described [27, 28]. For the BPRHS, fasting serum glucose and insulin were measured as previously described [29]. For both GOLDN and BPRHS, erythrocyte isolation and fatty acid extraction followed standard procedures [30, 31], and the final values of fatty acid methylesters were expressed as percentage of total fatty acids. Homeostasis model assessment of insulin resistance (HOMA-IR) (calculated as fasting insulin*fasting glucose/22.5) was used to assess insulin resistance. All glucose-related traits, including HOMA-IR, fasting insulin, glucose and adiponectin (GOLDN only) were Box-Cox [32] or log transformed to achieve normal distribution before analysis.

### Genome-wide genotyping

For GOLDN, genomic DNA was extracted and purified as described previously [33]. Affymetrix Genome-Wide Human SNP Array 6.0 (CA, USA) and the Birdseed calling algorithm were used to conduct genome-wide genotyping. A total number of 590,000 SNPs were selected for the genome-wide analysis in the present study according to the following criteria: call rate ≥96%, minor allele frequency (MAF) ≥5%, *P*-value ≥10^-6^ for the Hardy-Weinberg equilibrium (HWE) test, negligible Mendelian error within one family [24]. Briefly, SNPs were excluded if they were monomorphic or had a call rate < 96%. SNPs were also excluded based on the number of families with Mendel error: for MAF ≥20%, if errors were present in >3 families; for 20% > MAF ≥10%, if errors were present in >2 families; for 10% > MAF ≥5%, if errors were present in >1 family; and for MAF <5%, if any errors were present [24].

For the BPRHS, DNA was obtained from blood samples using QIAamp DNA Blood Mini Kits (Qiagen). GWAS genotyping was conducted using Affymetrix’s AxiomGenome-Wide LAT Array, which was designed especially for Hispanic populations and contains probe sets to genotype 817,810 SNPs. The Genome-Wide genotype was called and QC using Affymetrix^®^ Power Tools (APT), Genotyping Console (GTC), and R, following the standard protocols – Best Practices provided by the vender. Based on the criteria of SNPolisher, 804,947 SNPs passed general QC. Among them, 717,275 autosomal SNPs that met the following criteria: call rate ≥97%, minor allele frequency (MAF) ≥1%, *P*-value of Hardy-Weinberg Equilibrium (HWE) ≥10^-6^, were used in current study. To estimate population structure, 50,704 SNPs were further selected based on following criteria: MAF ≥5%, and pair-wise linkage disequilibrium R square ≤0.1. Using principle components analysis, as implemented in SVS (Golden Helix Inc., Bozeman, MT), we selected the first major principal component eigenvalue to present the population structure based on the scree plot. This was included in models for all the analyses to adjust for population structure.

### Genome-wide variance contribution of genotype by erythrocyte n-3 PUFA interaction to T2D-related traits in GOLDN and replication in BPRHS

For GOLDN, GxE variance contribution to the total variance of T2D-related traits was estimated using a tool for Genome-wide Complex Trait Analysis (GCTA) [34]. Within GCTA, the GxE interaction and main effects of genetic factors were treated as random effects in the model, with the main effects of E factors as fixed effects, while adjusting for potential confounders, including age, sex, body mass index, study center, energy intake, kinship and population structure. A “–gxe” option was used to estimate the variance contribution of GxE interaction in the GCTA. Briefly, GCTA was run in a Linux computer environment with the following steps: 1) generate three files (bed, bim and fam) for the GWAS genotypes using PLINK; 2) A “–make-grm” option was used to generate grm.gz and grm.id files; 3) prepare a phenotype file for each trait and a covariate file; 4) estimate the GxE variance contribution using a “-gxe” option. Based on the method of the GCTA power calculation by Visscher et al. [35], we calculated the power to estimate heritability greater than zero (h^2^ ≥ 0.2) to be 94% for GOLDN with family structures (n = 820, variance of the SNP-derived genetic relationships = 0.00093), 44% for the BPRHS participants without family structure (n = 716, variance of the SNP-derived genetic relationships = 0.00032).

An E factor is the environmental term in the GxE interaction, in this case the concentration of a particular PUFA in the erythrocyte membrane as a proxy for long-term dietary intake. Principle components analysis was used to calculate the population structure using SVS (Golden Helix Inc., Bozeman, MT.) [36, 37], and three key principle components were included in the model as covariates. GxE heritability was calculated as the GxE variance divided by the total phenotypic variance. In the GCTA, four T2D-related traits, including HOMA-IR, fasting insulin, glucose and adiponectin, were treated as phenotypes for the estimation of variance contribution. Eleven n-3 PUFAs or related E factors were available to this study: total n-3 PUFA, n-6 PUFA, ALA, EPA, DHA, EPA + DHA, docosapentaenoic acid (DPA, C22:5n3), arachidonic acid (AA, C20:4n6)/EPA, AA/DHA, AA/(EPA + DHA) and total n-6/n-3 PUFA ratio. Each E factor was categorized into quartiles for data analysis. As the ratio of n-6 by n-3 fatty acids was suggested to play an important role in the development of many chronic diseases [38], and different n-3 fatty acids may exert differential effects on the biological system [8, 15], we selected combinations of different n-6 by n-3 fatty acid ratios as the E factors. Bonferroni correction was employed to correct for multiple testing. In the GCTA analysis, the number of multiple tests was 44 (4 × 11), thus a *P*-value < 0.001 was considered as significant after correction (0.05/ (4 × 11)).

For BPRHS, GCTA was conducted to replicate the significant variance contribution produced by the GOLDN population. An E factor with non-significant variance contribution in the GOLDN was selected as a control for each trait. The potential confounders in BPRHS were age, sex, body mass index, energy intake and population structure. Principle components analysis was used to estimate the population structure using SVS (Golden Helix Inc., Bozeman, MT.) [36, 37].

### GxE genome-wide association study (GWAS) in GOLDN

Genome-wide GxE interaction analysis was conducted using linear mixed effects model (LME) under an additive genetic model in a GWAF package [39] in R, version 2.15.0. This method treats all genotypes and GxE interactions as fixed effects and the family relationship as a random effect through the kinship matrix. Quantile-quantile (QQ) plots were drawn in R to characterize the extent to which the observed GxE *P*-values follow the expected (null) distribution [40]. A *P*-value <1.0 × 10^-5^was considered significant, as this threshold is generally recommended for discovery in GWAS.

## Electronic supplementary material

Additional file 1: Table S1: GxE Variance contribution of erythrocyte n-3 polyunsaturated fatty acids to HOMA-IR. **Table S2.** GxE Variance contribution of erythrocyte n-3 polyunsaturated fatty acids to fasting insulin. **Table S3.** GxE Variance contribution of erythrocyte n-3 polyunsaturated fatty acids to fasting glucose. **Table S4.** GxE Variance contribution of erythrocyte n-3 polyunsaturated fatty acids to adiponectin. (DOC 98 KB)

Additional file 2: Table S5: SNP list identified from GxE GWAS for diabetes traits in GOLDN population. (XLSX 98 KB)

## Abbreviations

GxE: Genotype by environment
GCTA: Genome-wide Complex Trait Analysis
HOMA-IR: HOMA-Insulin Resistance
GWAS: Genome-wide association study
T2D: Type 2 diabetes
PUFA: Polyunsaturated fatty acids
DHA: Docosahexaenoic acid
EPA: Eicosapentaenoic acid
DPA: Docosapentaenoic acid
ALA: Alpha-linolenic acid
SNP: Single-nucleotide polymorphism
AA: Arachidonic acid
QQ: Quantile-quantile
GOLDN: Genetics of Lipid Lowering Drugs and Diet Network
BPRHS: Boston Puerto Rican Health Study.
