# Supplementary material for: Genome-wide interaction of genotype by erythrocyte n-3 fatty acids contributes to phenotypic variance of diabetes-related traits
Source: BMC Genomics. 2014 Sep 11;15(1):781. doi: 10.1186/1471-2164-15-781 (PMC4168207; doi:10.1186/1471-2164-15-781)
Supplement: Supplementary file 1 — Additional file 1: Table S1: GxE Variance contribution of erythrocyte n-3 polyunsaturated fatty acids to HOMA-IR. Table S2. GxE Variance contribution of erythrocyte n-3 polyunsaturated fatty acids to fasting insulin. Table S3. GxE Variance contribution of erythrocyte n-3 polyunsaturated fatty acids to fasting glucose. Table S4. GxE Variance contribution of erythrocyte n-3 polyunsaturated fatty acids to adiponectin. (DOC 98 KB) [file 12864_2014_6453_MOESM1_ESM.doc]

**Additional file 1: Table S1 GxE Variance contribution of erythrocyte n-3 polyunsaturated fatty acids to HOMA-IR1**

| E factor**^2^** | *P-*nominal (gxe) ^2^ | Vg | SE | Vgxe | SE | h^2^(g) (95%CI) | SE | h^2^(gxe) (95%CI) | SE | h^2^(g+gxe) (95%CI) |
| --- | --- | --- | --- | --- | --- | --- | --- | --- | --- | --- |
| Total n-3 PUFA | 0.191 | 0.0010 | 0.0005 | 0.0006 | 0.0008 | 17.6 (0.16, 35.0) | 8.9 | 11.5 (0, 38.5) | 13.8 | 29.1 (6.85, 51.3) |
| Total n-6 PUFA | 0.136 | 0.0009 | 0.0005 | 0.0008 | 0.0008 | 17.2 (0, 34.6) | 8.9 | 14.7 (0, 42.1) | 14.0 | 31.8 (9.36, 54.2) |
| n-6/ n-3 PUFA | 0.369 | 0.0011 | 0.0005 | 0.0003 | 0.0008 | 19.5 (2.25, 36.7) | 8.8 | 4.7 (0, 32.5) | 14.2 | 24.2 (1.66, 46.7) |
| EPA+DHA | 0.500 | 0.0010 | 0.0005 | 0.0000 | 0.0008 | 18.9 (2.04, 35.8) | 8.6 | 0.0 (0, 28) | 14.3 | 18.9 (0, 41.3) |
| ALA | 0.500 | 0.0011 | 0.0005 | 0.0000 | 0.0007 | 19.2 (2.34, 36.1) | 8.6 | 0.0 (0, 26.7) | 13.6 | 19.2 (0, 41.0) |
| DHA | 0.276 | 0.0011 | 0.0005 | 0.0004 | 0.0007 | 19.5 (2.45, 36.6) | 8.7 | 7.7 (0, 34.4) | 13.6 | 27.1 (5.25, 49.0) |
| EPA | 0.355 | 0.0011 | 0.0005 | 0.0003 | 0.0008 | 19.4 (2.15, 36.6) | 8.8 | 5.2 (0, 32.6) | 14.0 | 24.5 (2.16, 46.8) |
| **DPA** | **0.034** | **0.0007** | **0.0005** | **0.0015** | **0.0008** | **12.8 (0, 30.6)** | **9.1** | **26.5 (0, 55.7)** | **14.9** | **39.4 (15.9, 62.9)** |
| AA/ (EPA+DHA) | 0.399 | 0.0012 | 0.0005 | 0.0002 | 0.0008 | 21.1 (3.4, 13.7) | 8.7 | 3.4 (0, 30.3) | 13.7 | 24.5 (2.55, 46.5) |
| AA/ EPA | 0.500 | 0.0011 | 0.0005 | 0.0000 | 0.0008 | 19.9 (2.06, 37.7) | 9.1 | 0.0 (0, 27.4) | 14.0 | 19.9 (0, 42.5) |
| AA/ DHA | 0.500 | 0.0012 | 0.0005 | 0.0000 | 0.0007 | 21.3 (4.44, 38.2) | 8.6 | 0.0 (0, 26.3) | 13.4 | 21.3 (0, 42.9) |

^1^Without GxE: phenotypic variance of HOMA-IR, Vp=0.0056 (0.0003), Vg=0.0013 (0.0004), h^2^ (g)=23.4% (7.7%), *P*-value (g)=0.0004. PUFA, polyunsaturated fatty acid; DHA, docosahexaenoic acid; EPA, eicosapentaenoic acid; DPA, docosapentaenoic acid; ALA, alpha -linolenic acid; AA, arachidonic acid;Vg, additive genetic variance; V(gxe), variance contributed by GxE interaction; SE, standard error; h^2^ (g), additive genetic heritability; h^2^ (gxe), heritability explained by GxE interaction; h^2^ (g+gxe), total heritability. GxE heritability was calculated as the GxE variance divided by the total phenotypic variance.

^2^ *P*-value (gxe) of GxE interaction was adjusted for age, sex, body mass index, study center, energy intake, kinship, and population structure.

**Additional file 1: Table S2 GxE Variance contribution of erythrocyte n-3 polyunsaturated fatty acids to fasting insulin**1

| E factor**^2^** | *P-*nominal (gxe) ^2^ | Vg | SE | Vgxe | SE | h^2^(g) (95%CI) | SE | h^2^(gxe) (95%CI) | SE | h^2^(g+gxe) (95%CI) |
| --- | --- | --- | --- | --- | --- | --- | --- | --- | --- | --- |
| Total n-3 PUFA | 0.113 | 0.0003 | 0.0002 | 0.0005 | 0.0004 | 11.4 (0, 28.5) | 8.7 | 17.8 (0, 46.6) | 14.7 | 29.1 (6.17, 52.0) |
| Total n-6 PUFA | 0.074 | 0.0003 | 0.0002 | 0.0005 | 0.0004 | 11.5 (0, 28.4) | 8.6 | 20.7 (0, 49.3) | 14.6 | 32.2 (9.46, 54.9) |
| n-6/ n-3 PUFA | 0.440 | 0.0004 | 0.0002 | 0.0001 | 0.0004 | 15.4 (0, 32.3) | 8.6 | 2.3 (0, 31.1) | 14.7 | 17.8 (0, 40.6) |
| EPA+DHA | 0.500 | 0.0004 | 0.0002 | 0.0000 | 0.0004 | 16.0 (0, 32.7) | 8.5 | 0.0 (0, 27.8) | 14.2 | 16.0 (0, 38.2) |
| ALA | 0.500 | 0.0003 | 0.0002 | 0.0000 | 0.0004 | 12.8 (0, 28.9) | 8.2 | 0.0 (0, 27.2) | 13.9 | 12.8 (0, 34.5) |
| DHA | 0.231 | 0.0003 | 0.0002 | 0.0003 | 0.0004 | 13.5 (0, 30.4) | 8.6 | 10.0 (0, 37.6) | 14.1 | 23.5 (1.25, 45.7) |
| EPA | 0.234 | 0.0003 | 0.0002 | 0.0003 | 0.0004 | 12.9 (0, 30) | 8.7 | 10.5 (0, 38.9) | 14.5 | 23.5 (0.76, 46.2) |
| **DPA** | **0.042** | **0.0002** | **0.0002** | **0.0006** | **0.0004** | **8.6 (0, 25.8)** | **8.8** | **24.3 (0, 53.1)** | **14.7** | **32.9 (9.87, 55.9)** |
| AA/ (EPA+DHA) | 0.500 | 0.0004 | 0.0002 | 0.0000 | 0.0004 | 15.9 (0, 32.8) | 8.6 | 0.0 (0, 27.0) | 14.0 | 15.9 (0, 38.0) |
| AA/ EPA | 0.500 | 0.0004 | 0.0002 | 0.0000 | 0.0004 | 16.0 (0, 33.2) | 8.8 | 0.0 (0, 27.0) | 13.8 | 16.0 (0, 38.1) |
| AA/ DHA | 0.500 | 0.0004 | 0.0002 | 0.0000 | 0.0004 | 14.6 (0, 31.3) | 8.5 | 0.0 (27.6, 27.6) | 14.1 | 14.6 (0, 36.7) |

^1^Without GxE: phenotypic variance of fasting insulin, Vp=0.0026 (0.0001), Vg=0.0004 (0.0002), h^2^ (g)=17.3% (7.4%), *P*-value (g)=0.005. PUFA, polyunsaturated fatty acid; DHA, docosahexaenoic acid; EPA, eicosapentaenoic acid; DPA, docosapentaenoic acid; ALA, alpha -linolenic acid; AA, arachidonic acid;Vg, additive genetic variance; V(gxe), variance contributed by GxE interaction; SE, standard error; h^2^ (g), additive genetic heritability; h^2^ (gxe), heritability explained by GxE interaction; h^2^ (g+gxe), total heritability. GxE heritability was calculated as the GxE variance divided by the total phenotypic variance.

^2^ *P*-value (gxe) of GxE interaction was adjusted for age, sex, body mass index, study center, energy intake, kinship, and population structure.

**Additional file 1: Table S3 GxE Variance contribution of erythrocyte n-3 polyunsaturated fatty acids to fasting glucose**1

| E factor**^2^** | *P-*nominal (gxe) ^2^ | Vg | SE | Vgxe | SE | h^2^(g) (95%CI) | SE | h^2^(gxe) (95%CI) | SE | h^2^(g+gxe) (95%CI) |
| --- | --- | --- | --- | --- | --- | --- | --- | --- | --- | --- |
| Total n-3 PUFA | 0.383 | 0.6202 | 0.3072 | 0.1318 | 0.4600 | 18.1 (0.85, 35.3) | 8.8 | 3.8 (0, 30.1) | 13.4 | 21.9 (0.14, 43.7) |
| Total n-6 PUFA | 0.500 | 0.6764 | 0.3053 | 0.0025 | 0.4618 | 19.7 (2.65, 36.8) | 8.7 | 0.1 (0, 26.4) | 13.4 | 19.7 (0, 41.4) |
| n-6/ n-3 PUFA | 0.129 | 0.5259 | 0.3021 | 0.4931 | 0.4719 | 15.3 (0, 32.4) | 8.7 | 14.4 (0, 41.3) | 13.7 | 29.7 (7.75, 51.7) |
| EPA+DHA | 0.223 | 0.5485 | 0.2986 | 0.3211 | 0.4567 | 16.1 (0, 33.0) | 8.6 | 9.4 (0, 35.5) | 13.3 | 25.5 (4.04, 47.0) |
| ALA | 0.500 | 0.6411 | 0.3020 | 0.0000 | 0.4699 | 18.8 (1.75, 35.9) | 8.7 | 0.0 (0, 27.0) | 13.8 | 18.8 (0, 40.9) |
| DHA | 0.241 | 0.5864 | 0.3014 | 0.3110 | 0.4659 | 17.1 (0.24, 34.0) | 8.6 | 9.1 (0, 35.8) | 13.6 | 26.2 (4.44, 48.0) |
| EPA | 0.500 | 0.6589 | 0.3055 | 0.0000 | 0.4516 | 19.4 (2.15, 36.6) | 8.8 | 0.0 (0, 26.1) | 13.3 | 19.4 (0, 41.1) |
| DPA | 0.500 | 0.5659 | 0.2949 | 0.0000 | 0.4722 | 16.7 (0, 33.6) | 8.6 | 0.0 (0, 27.4) | 13.9 | 16.7 (0, 38.8) |
| **AA/ (EPA+DHA)** | **0.023** | **0.4518** | **0.3076** | **0.9295** | **0.4979** | **13.1 (0, 30.3)** | **8.8** | **27.0 (-1.03, 55.0)** | **14.3** | **40.2 (17.6, 62.8)** |
| AA/ EPA | 0.337 | 0.5877 | 0.3138 | 0.1861 | 0.4622 | 17.2 (0, 34.8) | 9.0 | 5.4 (0, 31.9) | 13.5 | 22.6 (0.55, 44.7) |
| AA/ DHA | 0.279 | 0.6157 | 0.2992 | 0.2391 | 0.4449 | 18.0 (1.34, 34.7) | 8.5 | 7.0 (0, 32.5) | 13.0 | 24.9 (3.83, 46.0) |

^1^Without GxE: phenotypic variance of fasting glucose, Vp=3.41 (0.17), Vg=0.70 (0.27), h^2^ (g)=20.6% (7.6%), *P*-value (g)=0.002. PUFA, polyunsaturated fatty acid; DHA, docosahexaenoic acid; EPA, eicosapentaenoic acid; DPA, docosapentaenoic acid; ALA, alpha -linolenic acid; AA, arachidonic acid;Vg, additive genetic variance; V(gxe), variance contributed by GxE interaction; SE, standard error; h^2^ (g), additive genetic heritability; h^2^ (gxe), heritability explained by GxE interaction; h^2^ (g+gxe), total heritability. GxE heritability was calculated as the GxE variance divided by the total phenotypic variance.

^2^ *P*-value (gxe) of GxE interaction was adjusted for age, sex, body mass index, study center, energy intake, kinship, and population structure.

**Additional file 1: Table S4 GxE Variance contribution of erythrocyte n-3 polyunsaturated fatty acids to adiponectin**1

| E factor**^2^** | *P-*nominal (gxe) ^2^ | Vg | SE | Vgxe | SE | h^2^(g) (95%CI) | SE | h^2^(gxe) (95%CI) | SE | h^2^(g+gxe) (95%CI) |
| --- | --- | --- | --- | --- | --- | --- | --- | --- | --- | --- |
| Total n-3 PUFA | 0.229 | 0.0218 | 0.0044 | 0.0043 | 0.0058 | 48.3 (31.2, 65.4) | 8.7 | 9.6 (0, 34.5) | 12.7 | 57.9 (36.9, 78.9) |
| Total n-6 PUFA | 0.500 | 0.0220 | 0.0043 | 0.0000 | 0.0057 | 48.9 (32.6, 65.2) | 8.3 | 0.0 (0, 24.9) | 12.7 | 48.9 (28.3, 69.5) |
| n-6/ n-3 PUFA | 0.190 | 0.0209 | 0.0044 | 0.0053 | 0.0059 | 46.7 (29.6, 63.8) | 8.7 | 11.9 (0, 37.8) | 13.2 | 58.6 (37.1, 80.1) |
| EPA+DHA | 0.500 | 0.0219 | 0.0044 | 0.0000 | 0.0058 | 48.6 (31.7, 65.5) | 8.6 | 0.0 (0, 25.1) | 12.8 | 48.6 (27.6, 69.6) |
| ALA | 0.500 | 0.0214 | 0.0044 | 0.0000 | 0.0056 | 48.2 (30.8, 65.6) | 8.9 | 0.0 (0, 24.7) | 12.6 | 48.2 (27.1, 69.3) |
| DHA | 0.500 | 0.0215 | 0.0044 | 0.0000 | 0.0059 | 47.9 (30.7, 65.1) | 8.8 | 0.0 (0, 25.7) | 13.1 | 47.9 (26.4, 69.4) |
| EPA | 0.080 | 0.0207 | 0.0043 | 0.0070 | 0.0054 | 45.8 (29.1, 62.5) | 8.5 | 15.4 (0, 38.5) | 11.8 | 61.3 (41.4, 81.2) |
| DPA | 0.500 | 0.0219 | 0.0043 | 0.0000 | 0.0055 | 48.7 (31.8, 65.6) | 8.6 | 0.0 (0, 24.1) | 12.3 | 48.7 (28.2, 69.2) |
| AA/ (EPA+DHA) | 0.098 | 0.0202 | 0.0045 | 0.0068 | 0.0056 | 44.8 (27.4, 62.2) | 8.9 | 15.0 (0, 39.3) | 12.4 | 59.8 (38.9, 80.7) |
| **AA/ EPA** | **0.058** | **0.0200** | **0.0044** | **0.0072** | **0.0052** | **44.4 (27.3, 61.5)** | **8.7** | **16.0 (0, 38.3)** | **11.4** | **60.4 (40.7, 80.1)** |
| AA/ DHA | 0.175 | 0.0209 | 0.0045 | 0.0049 | 0.0055 | 46.3 (29.1, 63.5) | 8.8 | 10.9 (0, 35.0) | 12.3 | 57.2 (36.5, 77.9) |

^1^Without GxE: phenotypic variance of adiponectin, Vp=0.045 (0.002), Vg=0.021 (0.004), h^2^ (g)=47.5% (7.6%), *P*-value (g)=4.5E-13. PUFA, polyunsaturated fatty acid; DHA, docosahexaenoic acid; EPA, eicosapentaenoic acid; DPA, docosapentaenoic acid; ALA, alpha -linolenic acid; AA, arachidonic acid;Vg, additive genetic variance; V(gxe), variance contributed by GxE interaction; SE, standard error; h^2^ (g), additive genetic heritability; h^2^ (gxe), heritability explained by GxE interaction; h^2^ (g+gxe), total heritability. GxE heritability was calculated as the GxE variance divided by the total phenotypic variance.

^2^ *P*-value (gxe) of GxE interaction was adjusted for age, sex, body mass index, study center, energy intake, kinship, and population structure.
